# The Clinical Significance of Changes in the Expression Levels of MicroRNA-1 and Inflammatory Factors in the Peripheral Blood of Children with Acute-Stage Asthma

**DOI:** 10.1155/2018/7632487

**Published:** 2018-06-26

**Authors:** Man Tian, Ying Zhou, Haoyuan Jia, Xuming Zhu, Yubao Cui

**Affiliations:** ^1^Department of Respiratory, Children's Hospital of Nanjing Medical University, Nanjing, Jiangsu 210008, China; ^2^Department of Pediatrics Laboratory, Wuxi Children's Hospital, Wuxi 214023, China; ^3^Department of Clinical Laboratory, Wuxi People's Hospital Affiliated to Nanjing Medical University, Wuxi 214023, China

## Abstract

This study assessed the changes and clinical significance of microRNA-1 (miR-1) and inflammatory factors in the peripheral blood of children with acute-stage asthma. 100 children with acute-stage asthma (study group) and 100 healthy children (control group) were enrolled. For all enrolled children, the peripheral blood levels of miR-1, interleukin-4 (IL-4), IL-5, IL-8, tumor necrosis factor-alpha (TNF-*α*), and interferon-*γ* (IFN-*γ*) were measured. The relative expression levels of miR-1 and IFN-*γ* in the peripheral blood of children in the study group were significantly lower than those in the control group, whereas expression levels of IL-4, IL-5, IL-8, and TNF-*α* were significantly higher. Moreover, these levels changed to a greater extent in patients with severe disease (*P *< 0.05). Further analyses showed that the miR-1 expression level positively correlated with IFN-*γ* and negatively correlated with IL-4, IL-5, IL-8, and TNF-*α* expression levels (*P *< 0.05). ROC curve analysis to identify diagnostic specificity and sensitivity showed that, for diagnosing exacerbation in asthma, the area under the curve (AUC) for miR-1 was the highest (AUC = 0.900,* P *< 0.05) of all tested markers; this held true for diagnosing severe asthma as well (AUC = 0.977,* P *< 0.05). Compared to healthy children, children with acute-stage asthma had a low miR-1 expression level and a Th1/Th2 imbalance in their peripheral blood. The changes were closely related, became more exaggerated with an increase in disease severity, and could be used as auxiliary variables for diagnosing asthma exacerbation and evaluating disease severity.

## 1. Introduction

Bronchial asthma is a chronic airway hyperresponsive disease associated with complex etiology and unclear pathogenesis. Symptoms of bronchial asthma may manifest due to limited ventilation, airway constriction, airway remodeling, and other pathological changes. There are currently an estimated 150 million asthma patients around the world, but the prevalence rate has been increasing. Multiple factors contribute to the pathogenesis of this disease, but recent research indicates it may exhibit polygenetic inheritance. Consequently, research on the genetic susceptibility and gene regulatory factors of asthma has become prevalent in the research community [1–3].

Childhood asthma is especially concerning, because the long-term prognosis of children with asthma is generally poor. This recurrent disease can be classified into 4 stages: acute onset, chronic persistent, clinical remission, and other. The acute onset stage is the “attack” period. During this stage, the patient may show sudden clinical symptoms, along with dyspnea, and the patient's life may be at risk. Timely emergency treatment is required for severe acute asthma attacks. Identifying biomarkers that can aid asthma diagnosis or enable better prognostic predications may facilitate improved outcomes, particularly among children.

MicroRNAs (miRs) are small noncoding RNAs that can regulate gene expression at the posttranscriptional level. miRs therefore play important regulatory roles in various physiological processes as well as in the pathogenesis of a variety of diseases including cancers and diseases with autoimmune, endocrine, and metabolic origins. Hence, various microRNAs have been used as gene targets or biological markers for the clinical diagnosis and treatment of multiple pathologies.

MicroRNA-1 (miR-1) was originally thought to be specifically expressed in cardiomyocytes and involved in the development and progression of heart disease and was therefore studied in relation to cardiac pathologies [4–6]. Subsequent studies discovered that there were three distinct gene clusters of muscle expressed miRs: a cluster on chromosome 18 encoding miR-1-2 and miR-133a-1, a cluster on chromosome 20 encoding miR-1-1 and miR-133a-2, and a cluster on chromosome 6 encoding miR-133b and miR-206 [7]. The two miR-1 transcripts (miR-1-1 and miR-1-2) share the same mature products and are expressed in both the myocardium and skeletal muscle cells, whereas miR-206 is expressed predominately in skeletal muscle [8, 9].

Muscle miRs not only are closely associated with cardiac hypertrophy, myocardial infarction, and other heart diseases but also are involved in the pathogenesis of malignant tumors, autoimmune diseases, and fibrotic diseases [10, 11]. Downregulation of miR-1 can be protective [12, 13] or pathogenic [14–18] in smooth muscle or in tumors depending on the context. In addition, the muscle miRs miR-133-b and miR-206 are expressed in a subset of helper T cells, Th17 cells [19], suggesting that these factors may have a positive role in the development of specific subsets of immune cells.

Other T cells, particularly the Th1/Th2 cell subset, contribute to the pathogenesis and progression of bronchial asthma. Th1 cells secrete mainly IL-2 and IFN-*γ*, whereas Th2 cells secrete mainly IL4, IL-5, IL-10, and IL-13. Functional equilibrium between Th1 and Th2 cells is important in immune function and infection control. Th2 cell hyperfunction is closely associated with limited ventilation, tracheal inflammation, and tracheal hyperresponsiveness in asthma patients and is relevant to the pathogenesis and progression of bronchial asthma [20]. Analyzing the expression levels of inflammatory factors and the genetic factors such as microRNAs that regulate them is therefore important to illuminate the pathogenesis of this disease. In this study, we analyzed the changes in the expression levels of miR-1 and inflammatory factors in peripheral blood of children with acute-stage asthma and the corresponding clinical significance of these changes.

## 2. Materials and Methods

### 2.1. Clinical Data

100 children with acute-stage asthma who were treated in our hospital from June 2015 to October 2016 were included in the study group. All 100 children met the diagnosis standards described in the* Guidelines for Diagnosis and Prevention of Bronchial Asthma in Pediatric Group* (version 2008) and the* Guidelines for Diagnosis and Prevention of Bronchial Asthma in Pediatric Group* (version 2016) published by the Group of Respiratory, the Society of Pediatrics, and the Chinese Medical Association. Children with other accompanying respiratory diseases, autoimmune diseases, and dysfunctions of the heart, kidney, and or other vital organs were excluded from the study. Children with asthma who suffered from viral/bacterial infections or treated with adrenal cortex hormones or immunomodulators 2 months before inclusion were also excluded from the study. Among the 100 children with asthma enrolled, 46 were males and 54 were females. The average age was 5.8 ± 1.9 years (range: 2–10 years). There were 44 cases of mild asthma, 39 cases of moderate asthma, and 17 cases of severe asthma. For comparison, 100 healthy children who received physical examinations at the hospital during the same time period were enrolled in a control group. There were 48 male and 52 female children in the control group. The children in the control group aged from 2 to 9 years, with an average age of 5.6 ± 1.8 years. Clinical examinations were performed for all children in the control group to exclude children with bronchial asthma. Other exclusion criteria for the control group were the same as those for the study group. The parents or guardians of children in both groups signed a form indicating their informed consent. The protocol of the study was reviewed and approved by the medical ethics committee of the study hospital.

### 2.2. Measurements and Test Methods

Peripheral blood samples were collected from fasting children in both groups in the early morning. Blood samples were treated using the following protocols: plasma were separated and extracted for total RNA with TRIzol reagent (Invitrogen), and the RNA samples were kept at −70°C until tested. Subsequently, the cDNA was synthesized via reverse transcription using iScript cDNA synthesis kit (Bio-Rad, USA) in accordance with the manufacturer's instructions, and the relative expressions of miR-1 were detected and compared using real-time quantitative fluorescent PCR (q-PCR) on a 7900 HT Sequence Detection System (ABI, USA). U6 was used as an internal reference, with primer sequences shown in Table 1. The volume of the reaction system was 20 *μ*L with these reaction conditions: incubation at 95°C for 30 s followed by 40 cycles of denaturing for 15 s at 95°C, annealing for 20 s at 60°C, and extending for 60 s at 70°C. The cycle thresholds (Ct values) were collected to determine the difference (ΔCt) in Ct value between the microRNA and reference genes, and the 2^−ΔΔCt^ method was used to calculate the relative expression of miR-1. An enzyme-linked immunosorbent assay (ELISA) sandwich technique was used to test and compare the levels of plasma IL-4, IL-5, IL-8, TNF-*α*, and IFN-*γ* with Sunny ELISA Assay Kit (Mutisciences, Hangzhou, China) in children in both groups.

### 2.3. Statistical Method

A database was created and analyzed with SPSS 22.0 statistical software for Windows. Data were expressed in the form of the average ± standard deviation ($\overset{-}{x}\pm s$). A one-way analysis of variance was used to compare the study group(s) and control group, and the least significant difference (LSD) method was used for pairwise comparison. The correlation of miR-1 and inflammatory factor expression levels was determined using linear correlation analysis, and the correlation of disease severity and miR-1 level as well as inflammatory factor expression level was performed with Spearman's rank correlation analysis. Receiver operator characteristics (ROC) curves were used to compare the values of various variables for diagnosing asthma and evaluating asthma severity. Using the area under the curve (AUC) as the basis, *P* < 0.05 indicated statistical differences.

## 3. Results

### 3.1. Expression Levels of miR-1 and Inflammatory Factors in Peripheral Blood of Children

Compared with the control group, the relative expression levels of miR-1 and IFN-*γ* in the peripheral blood of children with asthma in the study group were significantly decreased, whereas the expression levels of IL-4, IL-5, IL-8, and TNF-*α* were significantly increased. Changes in the variables increased gradually along with the severity of the disease (*P* < 0.05), as shown in Table 2.

### 3.2. Correlation between Expression Levels of miR-1 and Inflammatory Factors in Peripheral Blood of Children with Asthma

The miR-1 expression level in peripheral blood of children with asthma was positively correlated with the IFN-*γ* expression level (*P* < 0.05) and negatively correlated with the expression levels of IL-4, IL-5, IL-8, and TNF-*α*  (*P* < 0.05), as shown in Table 3.

### 3.3. Role of Peripheral Blood miR-1 and Inflammatory Factors in Diagnosing Asthma Attacks and Severity

**Figure 1:**
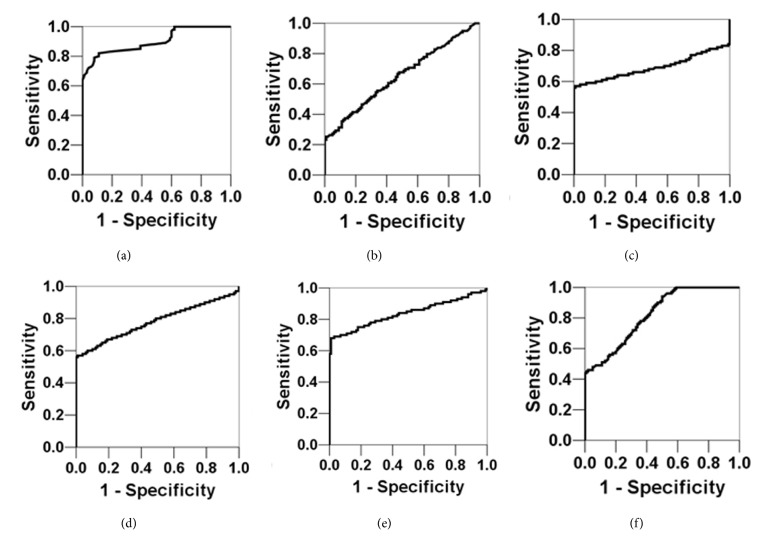
ROC curves of miR-1 and inflammatory factor expression levels in peripheral blood for diagnosing acute asthma attacks. (a) miR-1; (b) IL-4; (c) IL-5; (d) IL-8; (e) TNF-*α*; (f) IFN-*γ*. Of these markers, miR-1 has the highest AUC, demonstrating that it has the best accuracy in diagnosing acute asthma.

**Figure 2:**
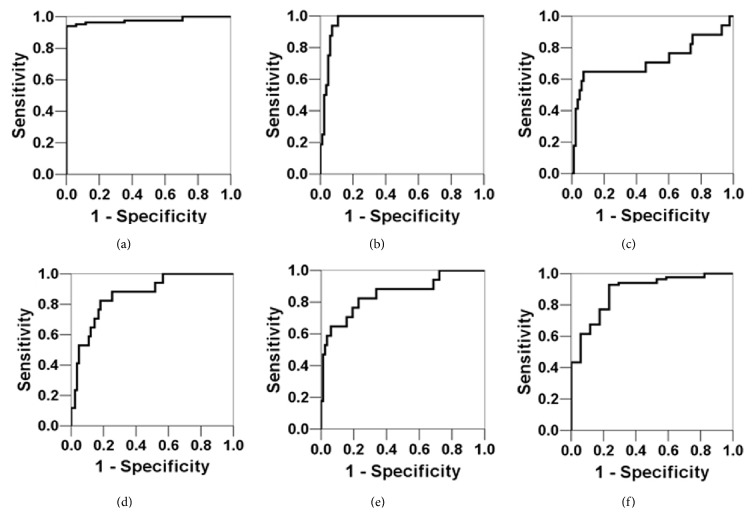
ROC curves of miR-1 and inflammatory factor expression levels in peripheral blood for diagnosing asthma severity. (a) miR-1; (b) IL-4; (c) IL-5; (d) IL-8; (e) TNF-*α*; (f) IFN-*γ*. Of the markers tested, the AUC was highest for miR-1, demonstrating that it has the best accuracy for diagnosing asthma severity.

We next employed an ROC curve analysis to determine the diagnostic utility of these measured circulating markers. ROC analysis is used to compare sensitivity and specificity for a diagnostic tool, and the AUC identifies its accuracy; the greater the area, the higher the accuracy. For diagnosing acute asthma attacks, the AUC of the miR-1 expression level was the highest (AUC = 0.900,* P* < 0.05), followed by the TNF-*α* level (AUC = 0.837,* P* < 0.05) and the IFN-*γ* level (AUC = 0.823,* P* < 0.05) (Table 4 and Figure 1). For diagnosing asthma severity, the AUC of the miR-1 expression level was the highest (AUC = 0.977, *P* < 0.05), followed by the IL-4 level (AUC = 0.963,* P* < 0.05) and the IFN-*γ* level (AUC = 0.885, *P* < 0.05) (Table 5 and Figure 2).

## 4. Discussion

In this study, the levels of miR-1 and the Th1-expressed cytokine IFN-*γ* in peripheral blood of children with acute-stage asthma were significantly lower, whereas the expression levels of the Th2 cytokines IL-4, IL-5, IL-8, and TNF-*α* were significantly higher than levels in children without asthma. These changes indicate an imbalance of Th1/Th2 function in children with acute-stage asthma. Such an imbalance could promote the diffusion and amplification of inflammatory reactions in the body. The changes in the serum markers tested increased gradually with exacerbation of the disease, indicating that the downregulation of miR-1 expression and Th1/Th2 dysfunction may play important roles in the progression of acute asthma. Since the levels of miR-1 and the various inflammatory factors were correlated, downregulation of miR-1 may directly affect Th2 cell hyperfunction and Th1 cell dysfunction, thereby playing an important role in inducing and enhancing immune system imbalance. Further studies are needed to clarify and better define the impact these changes have on asthma pathogenesis. Nonetheless, this study demonstrates that the miR-1 expression level in peripheral blood of children with acute-stage asthma may be an auxiliary marker for evaluating asthmatic attacks and disease severity.

MicroRNA expression is responsive to a variety of external factors [21–23] and closely coupled to the onset and progression of pathologies [24] including asthma. The expression levels of multiple miRNAs are known to be altered in children with asthma [25], but only two studies [26, 27] have specifically examined miR-1. Like this study, both reported that circulating miR-1 levels were reduced in asthma patients. It has been noted that the expression levels of other muscle-associated miRs are also affected in asthma. miR-133 and miR-206 were found to be altered in the peripheral blood of adults with asthma [28]. miRs, in particular the muscle miRs, may function in asthma by regulating bronchial smooth muscle cells [21] or pathways relating to smooth muscle cell such as Jak/STAT [29]. Downregulation of miR-133a, a muscle miR cocistronic with miR-1, in bronchial smooth muscle correlated with an increase in RhoA and an increase in bronchial smooth muscle contractility associated with airway hyperresponsiveness [30].

Muscle miRs also function in additional cells types. miR-133b and miR-206 are expressed during Th17 cell differentiation concomitant with the expression of IL-17 [19]. The Th17 lineage, like the Th2 lineage, is implicated in asthma and both lineages expand if Th1 differentiation is blocked [31]. Hence, muscle miRs could also function during inflammation by directly altering the balance of T cell subtypes, thus affecting the expression of Th-related cytokines. The direct targets of muscle miRs, including miR-1, have not been defined in immune cell types, but cytokine mRNAs are known to be targets of other miRs [32, 33].

In this study, there was a correlation between decreased levels of peripheral blood miR-1 and increased IL-4, IL-5, IL-8, and TNF-*α* and decreased IFN-*γ*, suggesting a direct or indirect effect of miR-1 on cytokine producing T cells. However, circulating miRs can affect target gene expression in multiple cell types in an autocrine manner [33]; hence, other cell lineages could also be affected. miRs are known to regulate gene expression in mast cells, eosinophils, macrophages, neutrophils, and airway epithelial cells to affect the secretion of inflammatory factors characteristic of asthma [33]. The complex web of interactions underlying the molecular biology of miRs in these cell types remains unclear; however, it is clear that circulating miRs are useful diagnostic markers, and if their levels can be manipulated in peripheral blood, they may offer a systematic approach for controlling asthma. Adding the detection of miR-1 circulating levels as a complementary approach in asthma diagnosis and management may also facilitate monitoring of disease progression. Further research is necessary to determine whether this marker may facilitate earlier diagnosis or better prognostication

In summary, the miR-1 expression level in the peripheral blood of children with acute-stage asthma was significantly decreased, along with an imbalance in the expression of Th1/Th2 inflammatory factors. The miR-1 level inversely correlated with disease severity and could be used as an auxiliary index for diagnosing and evaluating acute-stage asthma.

## Figures and Tables

**Table 1:** Primer sequences for q-PCR.

Primer	Sequence
miR-1	Forward: 5′-GGGGTGGAATGTAAAGAA-3′
	Reverse: 5′-TGCGTGTCGTGGAGTC-3′

U6	Forward: 5′-GCTTCGGCAGCACATATACTAAAAT-3′
	Reverse: 5′-CGCTTCACGAATTTGCGTGTCAT-3′

**Table 2:** Expression levels of miR-1 and inflammatory factors in peripheral blood.

Groups (disease severity)	N	miR-1 (2^−ΔΔCt^)	IL-4 (ng/L)	IL-5 (ng/L)	IL-8 (ng/L)	TNF-*α* (ng/L)	IFN-*γ* (ng/L)
Control	100	6.49 ± 2.04	75.49 ± 48.53	15.94 ± 4.64	708.60 ± 348.14	175.58 ± 96.51	84.55 ± 16.88
Study (mild)	44	4.05 ± 0.81^*∗*^	77.08 ± 45.60	20.48 ± 13.02	1201.53 ± 687.26^*∗*^	432.74 ± 257.66^*∗*^	70.25 ± 10.95^*∗*^
Study (medium)	39	3.25 ± 1.40^#&^	108.55 ± 59.89^#&^	28.61 ± 14.68^#&^	1592.93 ± 974.32^#&^	527.39 ± 327.63^&^	62.60 ± 13.22^#&^
Study (severe)	17	0.60 ± 0.51^*∗*#&^	189.89 ± 14.92^*∗*#&^	35.54 ± 18.59^*∗*#&^	2618.91 ± 685.81^*∗*#&^	913.59 ± 299.64^*∗*&^	48.26 ± 8.35^*∗*#&^
*F*		88.561	28.268	23.778	55.026	69.847	44.610
*P*		0.000	0.000	0.001	0.000	0.000	0.000

^*∗*^ *P* < 0.05 compared to control; ^#^*P* < 0.05 compared to mild; ^&^*P* < 0.05 compared to medium. *F*: the value of ANOVA (analysis of variance).

**Table 3:** Correlation analysis of miR-1 and inflammatory factor in peripheral blood of children with asthma.

	IL-4	IL-5	IL-8	TNF-*α*	IFN-*γ*
*r*	−0.768	−0.660	−0.814	−0.807	0.856
*P*	0.001	0.001	0.001	0.001	0.001

**Table 4:** Expression levels of miR-1 and inflammatory factors for diagnosing acute asthma attacks.

Variables	AUC	Std. error	*P*	95% confidence interval	
				Lower bound	Upper bound
miR-1	0.900	0.022	0.001	0.857	0.943
IL-4	0.651	0.039	0.001	0.575	0.727
IL-5	0.690	0.042	0.001	0.607	0.772
IL-8	0.781	0.034	0.001	0.714	0.848
TNF-*α*	0.837	0.030	0.001	0.778	0.897
IFN-*γ*	0.823	0.028	0.001	0.768	0.878

**Table 5:** miR-1 and inflammatory factor expression levels for diagnosing asthma severity.

Variables	AUC	Std. error	*P*	95% confidence interval	
				Lower bound	Upper bound
miR-1	0.977	0.014	0.001	0.950	1.003
IL-4	0.963	0.017	0.001	0.929	0.997
IL-5	0.718	0.088	0.005	0.545	0.890
IL-8	0.864	0.046	0.001	0.774	0.953
TNF-*α*	0.853	0.057	0.001	0.740	0.965
IFN-*γ*	0.885	0.043	0.001	0.801	0.970

## Data Availability

The data used to support the findings of this study are available from the corresponding author upon request.
